# Supplementary material for: Rotavirus alphagastroenteritidis: Circulating Strains After the Introduction of the Rotavirus Vaccine (Rotarix®) in Luanda Province of Angola
Source: Viruses. 2025 Jun 17;17(6):858. doi: 10.3390/v17060858 (PMC12197630; doi:10.3390/v17060858)
Supplement: Supplementary file 1 [file viruses-17-00858-s001.zip › viruses-3645073-supplementary.pdf]

		1	2	3	4	5	6	7	8	9	10	11	12	13	14	15	16	17	18	19	20	21	22	23	24	25
1	RVA/Human-wt/AGO/DV114HKK/2021/G3P[6]		0.053	0.041	0.021	0.000	0.133	0.063	0.133	0.130	0.000	0.000	0.007	0.115	0.121	0.126	0.122	0.114	0.133	0.140	0.142	0.110	0.129	0.134	0.130	2.375
2	RVA/porcine/ITA/13404-7/2006/GNDP[6]	0.356		0.032	0.030	0.053	0.137	0.063	0.137	0.133	0.053	0.053	0.048	0.111	0.118	0.146	0.155	0.117	0.162	0.128	0.118	0.112	0.130	0.144	0.137	3.359
3	RVA/Human-wt/HUN/BP133800/2004/G4P[6]	0.270	0.202		0.030	0.041	0.104	0.058	0.104	0.108	0.041	0.041	0.041	0.106	0.093	0.113	0.122	0.104	0.124	0.115	0.117	0.094	0.117	0.119	0.111	2.091
4	RVA/swine/ITA/22104-7/2006/GNDP[6]	0.134	0.199	0.194		0.021	0.126	0.056	0.126	0.123	0.021	0.021	0.023	0.114	0.110	0.123	0.118	0.107	0.136	0.132	0.130	0.109	0.134	0.141	0.128	3.359
5	RVA/Human-wt/AGO/DV68HKK/2021/G3P[6]	0.000	0.356	0.270	0.134		0.133	0.063	0.133	0.130	0.000	0.000	0.007	0.115	0.121	0.126	0.122	0.114	0.133	0.140	0.142	0.110	0.129	0.134	0.130	2.375
6	RVA/Human-wt/AGO/DV105HMT/2021/G12P[8]	0.896	0.908	0.760	0.848	0.896		0.103	0.000	0.002	0.133	0.133	0.129	0.014	0.022	0.007	0.030	0.012	0.035	0.009	0.011	0.021	0.010	0.010	0.006	2.037
7	RVA/porcine/Gottfried/1990/GNDP[6]	0.435	0.417	0.377	0.369	0.435	0.725		0.103	0.100	0.063	0.063	0.071	0.098	0.101	0.107	0.104	0.097	0.107	0.107	0.109	0.099	0.103	0.103	0.104	3.437
8	RVA/Human-wt/AGO/DV117HMT/2021/G12P[8]	0.896	0.908	0.760	0.848	0.896	0.000	0.725		0.002	0.133	0.133	0.129	0.014	0.022	0.007	0.030	0.012	0.035	0.009	0.011	0.021	0.010	0.010	0.006	2.037
9	RVA/Human-wt/AGO/DV66HMZ/2022/G12P[8]	0.883	0.894	0.772	0.836	0.883	0.002	0.715	0.002		0.130	0.130	0.126	0.013	0.021	0.006	0.029	0.012	0.033	0.009	0.010	0.021	0.010	0.010	0.005	1.937
10	RVA/Human-wt/AGO/CDV120HKK/2021/GNDP[6]	0.000	0.356	0.270	0.134	0.000	0.896	0.435	0.896	0.883		0.000	0.007	0.115	0.121	0.126	0.122	0.114	0.133	0.140	0.142	0.110	0.129	0.134	0.130	2.375
11	RVA/Human-wt/AGO/DV177HKK/2021/G3P[6]	0.000	0.356	0.270	0.134	0.000	0.896	0.435	0.896	0.883	0.000		0.007	0.115	0.121	0.126	0.122	0.114	0.133	0.140	0.142	0.110	0.129	0.134	0.130	2.375
12	RVA/Human-wt/AGO/C27/2012/G1P[6]	0.025	0.318	0.260	0.137	0.025	0.871	0.457	0.871	0.859	0.025	0.025		0.111	0.118	0.122	0.123	0.110	0.134	0.135	0.138	0.107	0.124	0.130	0.126	3.044
13	RVA/Vaccine/USA/RotaTeq-WI79-4/1992/G6P[8]	0.807	0.779	0.749	0.786	0.807	0.079	0.691	0.079	0.077	0.807	0.807	0.785		0.020	0.013	0.028	0.005	0.031	0.014	0.015	0.019	0.016	0.015	0.012	2.131
14	RVA/Vaccine/USA/Rotarix-A41CB052A/1988/G1P[8]	0.833	0.792	0.684	0.764	0.833	0.136	0.713	0.136	0.133	0.833	0.833	0.809	0.124		0.021	0.028	0.019	0.027	0.019	0.019	0.005	0.020	0.022	0.020	4.143
15	RVA/Human-wt/USA/SSCRTV_00075/2015/G12P[8]	0.852	0.936	0.779	0.831	0.852	0.021	0.731	0.021	0.019	0.852	0.852	0.829	0.077	0.133		0.027	0.011	0.032	0.009	0.010	0.021	0.009	0.010	0.004	1.374
16	RVA/Human/BTN-49/BTN/2010/G9P[8]	0.824	0.979	0.834	0.821	0.824	0.199	0.738	0.199	0.195	0.824	0.824	0.826	0.181	0.184	0.183		0.026	0.006	0.031	0.031	0.025	0.027	0.031	0.029	4.694
17	RVA/Bethesda/DC140/1975/G3P[8]	0.795	0.817	0.738	0.752	0.795	0.062	0.692	0.062	0.059	0.795	0.795	0.773	0.013	0.118	0.059	0.167		0.029	0.012	0.013	0.018	0.013	0.013	0.011	1.756
18	RVA/Hu/IND/MMC38/2009/GNDP[8]	0.876	1.012	0.848	0.903	0.876	0.221	0.761	0.221	0.217	0.876	0.876	0.878	0.202	0.177	0.213	0.021	0.186		0.035	0.035	0.024	0.032	0.036	0.033	3.380
19	RVA/Hu/GER126-08/GER/2008/G12P[8]	0.927	0.881	0.797	0.876	0.927	0.037	0.737	0.037	0.035	0.927	0.927	0.900	0.077	0.120	0.035	0.200	0.060	0.222		0.005	0.019	0.009	0.009	0.007	1.996
20	RVA/Human-wt/FRA/E9779/2013/G1P[8]	0.936	0.836	0.817	0.872	0.936	0.050	0.744	0.050	0.048	0.936	0.936	0.910	0.087	0.119	0.048	0.198	0.069	0.220	0.015		0.018	0.010	0.010	0.009	2.106
21	RVA/Human-wt/USA/Wa/1974/G1P[8]	0.777	0.773	0.678	0.768	0.777	0.134	0.706	0.134	0.130	0.777	0.777	0.755	0.113	0.013	0.130	0.162	0.107	0.152	0.118	0.117		0.020	0.021	0.020	3.283
22	RVA/Human-wt/BRA/BA20142/2011/G12P[8]	0.877	0.874	0.801	0.881	0.877	0.044	0.719	0.044	0.041	0.877	0.877	0.852	0.085	0.124	0.041	0.177	0.067	0.201	0.037	0.046	0.122		0.006	0.008	1.597
23	RVA/Human-wt/AGO/CSP10/2013/G1P[8]	0.902	0.931	0.813	0.908	0.902	0.044	0.718	0.044	0.041	0.902	0.902	0.877	0.085	0.137	0.041	0.201	0.067	0.227	0.037	0.046	0.128	0.019		0.008	2.305
24	RVA/Human-wt/MWI/BID1LN/2013/G1P[8]	0.876	0.902	0.777	0.854	0.876	0.015	0.718	0.015	0.013	0.876	0.876	0.851	0.069	0.124	0.009	0.192	0.052	0.214	0.025	0.037	0.121	0.031	0.031		1.577
25	RVC/Hu/JPN/Y12-1/2012	5.635	6.278	5.188	6.069	5.635	4.905	6.717	4.905	4.769	5.635	5.635	6.120	4.874	6.574	4.101	7.199	4.502	6.448	4.936	5.056	6.065	4.352	5.080	4.321	

Figure S1. Nucleotide distance matrix for VP4 gene.

The number of base substitutions per site from between sequences are shown. Standard error estimate(s) are shown above the diagonal and were obtained by a bootstrap procedure (1000 replicates). The analysis involved 25 nucleotide sequences. There was a total of 563 positions. Evolutionary analyses were conducted in MEGA7.

		1	2	3	4	5	6	7	8	9	10	11	12	13	14	15	16	17	18	19	20	21	22	23	24	25	26	27	28	29
1	RVA/Human-wt/PHL/L26/1987/G12P[4]		0.069	0.069	0.026	0.070	0.066	0.075	0.022	0.069	0.069	0.077	0.092	0.025	0.023	0.024	0.095	0.066	0.089	0.073	0.098	0.074	0.068	0.071	0.059	0.067	0.079	0.078	0.067	0.078
2	RVA/Human-wt/AGO/DV34HECJ/2022/G8P[ND]	0.400		0.000	0.078	0.065	0.007	0.063	0.087	0.069	0.078	0.082	0.083	0.083	0.081	0.085	0.061	0.057	0.062	0.061	0.063	0.073	0.088	0.061	0.075	0.033	0.010	0.046	0.040	0.036
3	RVA/Human-wt/AGO/DV540HGL/2022/G8P[ND]	0.400	0.000		0.078	0.065	0.007	0.063	0.087	0.069	0.078	0.082	0.083	0.083	0.081	0.085	0.061	0.057	0.062	0.061	0.063	0.073	0.088	0.061	0.075	0.033	0.010	0.046	0.040	0.036
4	RVA/Human-wt/AGO/DV117HMT/2021/G12P[8]	0.127	0.439	0.439		0.060	0.072	0.069	0.035	0.058	0.071	0.075	0.075	0.012	0.012	0.012	0.081	0.056	0.082	0.061	0.084	0.067	0.066	0.057	0.070	0.068	0.075	0.068	0.075	0.082
5	RVA/Human-wt/AGO/DV72HMCC/2022/G9P[ND]	0.393	0.379	0.379	0.338		0.063	0.020	0.058	0.025	0.028	0.026	0.063	0.065	0.068	0.062	0.058	0.053	0.058	0.018	0.056	0.072	0.031	0.006	0.076	0.058	0.065	0.084	0.087	0.076
6	RVA/Human-wt/AGO/CSMI10/2012/G8P[6]	0.382	0.018	0.018	0.419	0.363		0.060	0.084	0.066	0.071	0.083	0.084	0.077	0.076	0.079	0.054	0.052	0.055	0.059	0.056	0.065	0.083	0.059	0.069	0.031	0.013	0.044	0.035	0.033
7	RVA/Human-wt/Malawi/MW47/1997/G9P[ND]	0.423	0.366	0.366	0.385	0.087	0.349		0.081	0.031	0.033	0.028	0.072	0.072	0.070	0.073	0.068	0.054	0.064	0.006	0.066	0.065	0.035	0.018	0.078	0.050	0.067	0.069	0.057	0.064
8	RVA/Porcine/IND/RU172/2002/G12P[ND]	0.102	0.477	0.477	0.175	0.328	0.455	0.445		0.058	0.066	0.073	0.088	0.031	0.033	0.028	0.090	0.071	0.091	0.075	0.093	0.079	0.064	0.059	0.078	0.070	0.095	0.112	0.087	0.095
9	RVA/Human-wt/CHN/97SZ37/1997/G9P[ND]	0.401	0.403	0.403	0.346	0.117	0.385	0.148	0.335		0.025	0.027	0.053	0.065	0.065	0.062	0.056	0.048	0.057	0.029	0.058	0.064	0.027	0.025	0.071	0.063	0.069	0.091	0.066	0.080
10	RVA/Human-wt/USA/F45/1987/G9P[ND]	0.399	0.427	0.427	0.401	0.140	0.387	0.168	0.369	0.116		0.024	0.061	0.074	0.075	0.073	0.066	0.055	0.062	0.031	0.068	0.082	0.029	0.029	0.067	0.062	0.081	0.081	0.072	0.079
11	RVA/Human-wt/USA/OM46/1997/G9P[8]	0.423	0.445	0.445	0.399	0.130	0.447	0.136	0.406	0.127	0.120		0.076	0.083	0.075	0.072	0.065	0.062	0.064	0.028	0.067	0.067	0.025	0.026	0.076	0.065	0.080	0.083	0.074	0.082
12	RVA/Simian-tc/USA/RRV/1975/G3P[3]	0.509	0.443	0.443	0.422	0.359	0.444	0.394	0.490	0.307	0.340	0.403		0.089	0.093	0.077	0.055	0.054	0.057	0.066	0.056	0.053	0.071	0.066	0.049	0.077	0.074	0.080	0.072	0.084
13	RVA/Human-wt/PV/2119/2011/G12P[ND]	0.125	0.456	0.456	0.048	0.374	0.435	0.414	0.146	0.392	0.421	0.442	0.487		0.013	0.008	0.086	0.063	0.086	0.064	0.089	0.064	0.064	0.062	0.064	0.068	0.085	0.072	0.078	0.087
14	RVA/Human-wt/PRY/Py1135ASR07/2007/G12P[9]	0.111	0.451	0.451	0.041	0.384	0.430	0.395	0.158	0.373	0.423	0.400	0.501	0.052		0.014	0.087	0.059	0.082	0.070	0.090	0.074	0.066	0.061	0.065	0.068	0.085	0.079	0.075	0.083
15	RVA/Human-wt/AGO/AH6/2012/G12P[6]	0.116	0.457	0.457	0.044	0.343	0.436	0.400	0.130	0.359	0.407	0.385	0.427	0.021	0.048		0.089	0.063	0.090	0.065	0.093	0.062	0.059	0.059	0.065	0.069	0.084	0.073	0.076	0.087
16	RVA/Human-wt/AGO/DV177HKK/2021/G3P[6]	0.500	0.358	0.358	0.454	0.328	0.325	0.370	0.484	0.326	0.380	0.355	0.305	0.475	0.477	0.472		0.021	0.004	0.059	0.003	0.069	0.070	0.060	0.064	0.061	0.059	0.064	0.068	0.065
17	RVA/Vaccine/USA/RotaTeq-WI78-8/1992/G3P[5]	0.387	0.325	0.325	0.336	0.313	0.310	0.317	0.407	0.289	0.336	0.346	0.295	0.373	0.352	0.364	0.087		0.019	0.050	0.021	0.073	0.067	0.055	0.056	0.056	0.060	0.064	0.062	0.060
18	RVA/Human-wt/AGO/DV114HKK/2021/G3P[6]	0.475	0.358	0.358	0.454	0.328	0.325	0.351	0.484	0.326	0.361	0.355	0.313	0.475	0.454	0.472	0.006	0.078		0.060	0.005	0.070	0.071	0.060	0.060	0.057	0.060	0.064	0.063	0.066
19	RVA/Human-wt/AGO/C84/2012/G9P[6]	0.414	0.357	0.357	0.348	0.078	0.341	0.012	0.413	0.138	0.157	0.136	0.365	0.375	0.396	0.362	0.334	0.302	0.334		0.062	0.064	0.033	0.018	0.076	0.049	0.065	0.071	0.059	0.067
20	RVA/Human-wt/AGO/DV68HKK/2021/G3P[6]	0.512	0.366	0.366	0.465	0.319	0.333	0.360	0.495	0.335	0.390	0.364	0.313	0.487	0.489	0.484	0.003	0.091	0.009	0.342		0.071	0.072	0.058	0.066	0.063	0.061	0.062	0.069	0.064
21	RVA/Dog-tc/USA/CU-1/1982/G3P[ND]	0.414	0.411	0.411	0.396	0.401	0.373	0.369	0.444	0.367	0.431	0.378	0.279	0.377	0.417	0.363	0.365	0.392	0.365	0.360	0.375		0.075	0.065	0.042	0.075	0.075	0.078	0.073	0.064
22	RVA/Human-wt/IND/116E/1986/G9P[11]	0.391	0.478	0.478	0.364	0.153	0.457	0.166	0.361	0.123	0.142	0.111	0.387	0.363	0.364	0.332	0.384	0.384	0.384	0.154	0.394	0.411		0.028	0.087	0.069	0.092	0.106	0.084	0.106
23	RVA/Human-wt/VNM/VE17426/2020/G9P[8]	0.402	0.370	0.370	0.330	0.012	0.354	0.078	0.336	0.117	0.150	0.130	0.378	0.364	0.356	0.333	0.336	0.321	0.336	0.078	0.327	0.373	0.142		0.079	0.058	0.061	0.086	0.077	0.073
24	RVA/Rabbit-tc/ITA/30-96/1996/G3P[14]	0.360	0.414	0.414	0.418	0.420	0.394	0.416	0.447	0.389	0.369	0.426	0.266	0.398	0.390	0.393	0.349	0.320	0.331	0.407	0.358	0.207	0.454	0.430		0.075	0.080	0.082	0.061	0.070
25	RVA/Human-wt/THA/TM-12505_60/2018/G8P[8]	0.389	0.167	0.167	0.396	0.341	0.156	0.304	0.398	0.372	0.366	0.372	0.435	0.390	0.396	0.391	0.365	0.340	0.348	0.297	0.374	0.424	0.380	0.350	0.427		0.031	0.038	0.039	0.042
26	RVA/Human-tc/MWI/QEC287/2006/G8P[8]	0.458	0.030	0.030	0.443	0.379	0.044	0.393	0.518	0.402	0.448	0.434	0.416	0.483	0.478	0.472	0.348	0.341	0.348	0.384	0.357	0.411	0.502	0.370	0.434	0.170		0.040	0.038	0.041
27	RVA/Human-wt/Croatia/CR2006/2006/G8P[8]	0.422	0.239	0.239	0.387	0.444	0.225	0.388	0.546	0.477	0.448	0.435	0.432	0.402	0.430	0.393	0.343	0.345	0.343	0.399	0.334	0.416	0.518	0.455	0.440	0.201	0.212		0.040	0.055
28	RVA/Human-wt/ITA/PR1300/2004/G8P[14]	0.381	0.206	0.206	0.413	0.458	0.183	0.327	0.461	0.378	0.402	0.404	0.413	0.430	0.404	0.409	0.391	0.365	0.372	0.336	0.401	0.412	0.446	0.424	0.366	0.214	0.210	0.229		0.032
29	RVA/Human-wt/AUS/BG8.01/1996/G8P[ND]	0.437	0.195	0.195	0.450	0.419	0.178	0.357	0.502	0.428	0.418	0.426	0.471	0.468	0.452	0.457	0.381	0.339	0.381	0.366	0.372	0.374	0.534	0.408	0.400	0.235	0.220	0.305	0.158	

Figure S2. Nucleotide distance matrix for VP7 gene.

The number of base substitutions per site from between sequences are shown. Standard error estimate(s) are shown above the diagonal and were obtained by a bootstrap procedure (1000 replicates). The analysis involved 29 nucleotide sequences. There was a total of 354 positions. Evolutionary analyses were conducted in MEGA7.
